# Acupuncture for the treatment of anxiety and depression in patients with spinal cord injury: A study protocol for systematic review and meta analysis

**DOI:** 10.1097/MD.0000000000039701

**Published:** 2024-09-20

**Authors:** Ke Liu, Xiaoyong Gao, Liang Ou, Zuyu Tang, Haoming Zhao, Sheng Hua, Yixiao Xiong, Le Zhang, Jianjun Kuang

**Affiliations:** a Hunan University of Chinese Medicine, Changsha, China; b Affiliated Hospital of Shanxi University of Traditional Chinese Medicine, Taiyuan, China; c Hunan Academy of Chinese Medicine, Changsha, China; d The Affiliated Hospital of Hunan Academy of Traditional Chinese Medicine, Changsha, China.

**Keywords:** acupuncture, anxiety, depression, meta-analysis, spinal cord injury, systematic review

## Abstract

**Background:**

Spinal cord injury patients frequently suffer from anxiety and depression, which can seriously affect their quality of life and recovery. Acupuncture, as a traditional Chinese therapy, has been used to treat anxiety and depression for more than 2000 years. The aim is to evaluate the clinical efficacy of acupuncture in the treatment of anxiety and depression in spinal cord injury patients.

**Methods:**

The literature on acupuncture treating anxiety and depression in patients with spinal cord injury in PubMed, Embase, Cochrane Library, Chinese Biomedical Literature Database, China National Knowledge Infrastructure, Chinese Scientific Journal Data, and Wanfang data were searched through computers from the establishment of the database to May 2024. In the study, the Cochrane tool for assessing the risk of bias was used and the meta-analyses were carried out using the software package Review Manager 5.4.

**Results:**

Ten trials were included in this systematic review, with 361 cases in the experimental group and 355 cases in the control group. Meta-analysis showed that compared with conventional treatment, acupuncture combined with conventional treatment was beneficial in improving the total clinical efficacy (odds ratio = 3.55 [95% confidence interval {CI}: 1.34–9.37], *P* < .001). We found acupuncture-assisted therapy could be beneficial in improving the Modified Barthel Index (MD = 10.48 [95% CI: 4.78–16.19], *P* < .001) and reducing anxiety or depression scores (such as the Self-Rating Anxiety Scale [MD = −6.08 {95% CI: −6.85 to −5.30}, *P* < .001]; reducing the Self-Rating Depression Scale [MD = −6.01 {95% CI: −6.95 to −5.07}, *P* < .001]). In addition, the study showed that the application of acupuncture treatment could improve 5-hydroxytryptamine compared to control group (MD = 44.99 [95% CI: 40.04–49.95], *P* < .001) and reduce TNF-α compared to control group (MD = −7.78 [95% CI: −8.73 to −6.83], *P* < .001).

**Conclusion:**

Acupuncture could be used as a complementary therapy to reduce anxiety and depression in spinal cord injury patients. Further original and high-quality research is needed to verify the conclusions of this study.

## 1. Introduction

Spinal cord injury (SCI) is an injury to the spinal cord that results in temporary or permanent changes in its function and is characterized by high costs, high rates of disability, and low age of onset, as well as a severe psychological burden on patients.^[1]^ Global survey data show that the number of SCI has increased dramatically from 1990 to 2019, with an 81.5% increase in prevalence, and in 2019, there were already 20.6 million SCI patients worldwide.^[2]^ Depression is ranked as the fourth leading cause of disability worldwide.^[3]^ A survey showed that 84.3% of SCI patients were depressed. Among them, 60.8% had moderate to severe depressive mood,^[4]^ and post-SCI depression is not only associated with suicidality but in addition may interfere with the physical recovery process.^[5]^ Depression mediated the relationships between precentral-subcortical causal links and motor recovery in SCI patients and depression-induced persistent low mood and loss of interest in activities. Consequently, lack of motivation, makes a recovery significantly less likely and is one of the most important risk factors for suicide in patients.^[6–8]^ Currently, these mental disorders in this population are generally underestimated or neglected and are often intervened in clinical practice with medications, cognitive behavioral therapy, etc, which, however, do not show satisfactory results, with the side effects of anxiolytic/antidepressant treatments being unavoidable in long-term treatment.^[3]^

Given the severity of this problem, there is an increasing need to address these psychiatric disorders in patients with SCI, which is conducive to improving their prognosis. Acupuncture has been used for thousands of years for the prevention and treatment of spinal cord injuries and psychiatric disorders and has been clinically applied to treat psychiatric disorders with good efficacy.^[9]^ Acupuncture has been shown to have an antidepressant effect by regulating neurotransmitter levels, modulating the neuroendocrine axis, improving neuroplasticity, and anti-inflammatory effects, and improving mood states.^[10]^

Previous research on acupuncture for anxiety and depression has focused on comorbidities associated with medical conditions. The main indicators used in previous studies were anxiety and depression scales, and there was a lack of objective indicators, which made the results unreliable.^[11,12]^ In view of this, we assessed the effect of acupuncture on post-SCI depression by analyzing all currently available randomized controlled trials (RCTs) to provide more effective and reliable treatment recommendations.

## 2. Materials and methods

### 2.1. Protocol and registration

This meta-analysis has been registered in the International Prospective Register of Systematic Reviews. https://www.crd.york.ac.uk/PROSPERO/display_record.php?RecordID=549267, identifier CRD 42024549267.

### 2.2. Search strategy

Potentially eligible trials were searched in PubMed, Embase, Cochrane Central Register of Controlled Trials, Chinese Biomedical Literature Database, China National Knowledge Infrastructure, Chinese Scientific Journal Data, and Wanfang data, up to May 5, 2024. The search strategy uses medical subject’s headings terms combined with free text, for example, “Acupuncture Therapy,” “Acupuncture,” “Spinal Cord Injuries,” “Spinal Cord Contusion,” “depression,” “anxiety,” etc. The detailed search strategies are displayed in Supplementary Table 1, Supplemental Digital Content, http://links.lww.com/MD/N555.

### 2.3. Inclusion criteria

#### 2.3.1. Types of studies

RCTs related to the application of acupuncture for the treatment of anxiety and depression in patients with SCI were searched. There are no restrictions regarding language, year of publication, etc.

#### 2.3.2. Types of participants

Patients were diagnosed with SCI with anxiety and depression using internationally and nationally recognized diagnostic criteria.

#### 2.3.3. Intervention

The same conventional treatment was given to both the control and experimental groups. The conventional treatment includes medication, physiotherapy, psychotherapy, and so on. The experimental group received acupuncture therapy based on conventional treatment.

#### 2.3.4. Outcomes

The primary outcomes included the total clinical efficacy rate. The secondary outcomes included the Self-Rating Anxiety Scale; Self-Rating Depression Scale; Hamilton Depression Scale; Hamilton Anxiety Scale; 5-hydroxytryptamine (5-HT), tumor necrosis factor-α (TNF-α); and Modified Barthel Index.

### 2.4. Exclusion criteria

1. The experimental group included other interventions (e.g., Chinese herbs) for Traditional Chinese Medicine treatment.
2. Protocols, reviews, and animal studies.
3. Duplicate articles, full papers, or papers without data.
4. Studies with academic misconduct (including plagiarism and falsification of data) were excluded.

### 2.5. Literature screening and data extraction

Two investigators used a predesigned spreadsheet to separately extract the essential content from inclusion papers: lead author, year of publication, patient age and gender, type of intervention, fracture type, dosage, course of treatment, and outcomes. Any discrepancies in the cross-checking procedure were resolved through discussion. Otherwise, the dispute was the subject of arbitration by a third party and other researchers.

### 2.6. Risk of bias assessment

Following the standards recommended in the Cochrane manual (Higgins JPT, 2021), the methodological quality of all included literature was evaluated independently by 2 reviewers. Discussion with the third author resolved any discrepancies. The risk of bias for each trial was assessed from 7 perspectives: sequence generation, allocation concealment, participant and personnel blinding, outcome assessment blinding, incomplete outcome data, selective reporting, and other biases. There are 3 levels of risk: high, low, or unclear, based on the evaluation result for each item.

### 2.7. Statistical analysis

The Review Manager 5.4 was applied to all meta-analyses of observational indicators in the selected literatures, and the corresponding results were intuitively displayed on the forest plot. In this review, we used mean difference (MD) to pool continuous variables. If each original study outcome indicator unit is inconsistent, the standard mean difference alternative MD should be selected. Dichotomous variables were pooled using the odds ratio (OR). All pooling effects are reported with 95% confidence intervals (95% CI). A *P*-value of <0.05 was considered statistically significant. The test for heterogeneity was performed using the *I*^2^ statistic and the Cochran *Q* testing. High heterogeneity was indicated by an *I*^2^ statistic >50%. Fixed effects model is used for *I*^2^ statistic <50%, otherwise random-effects model is selected. Sensitivity analysis tested the stability of the results. Stata 14 was used to estimate publication bias using Egger tests.

## 3. Results

### 3.1. Literature search results

**Figure 1.**
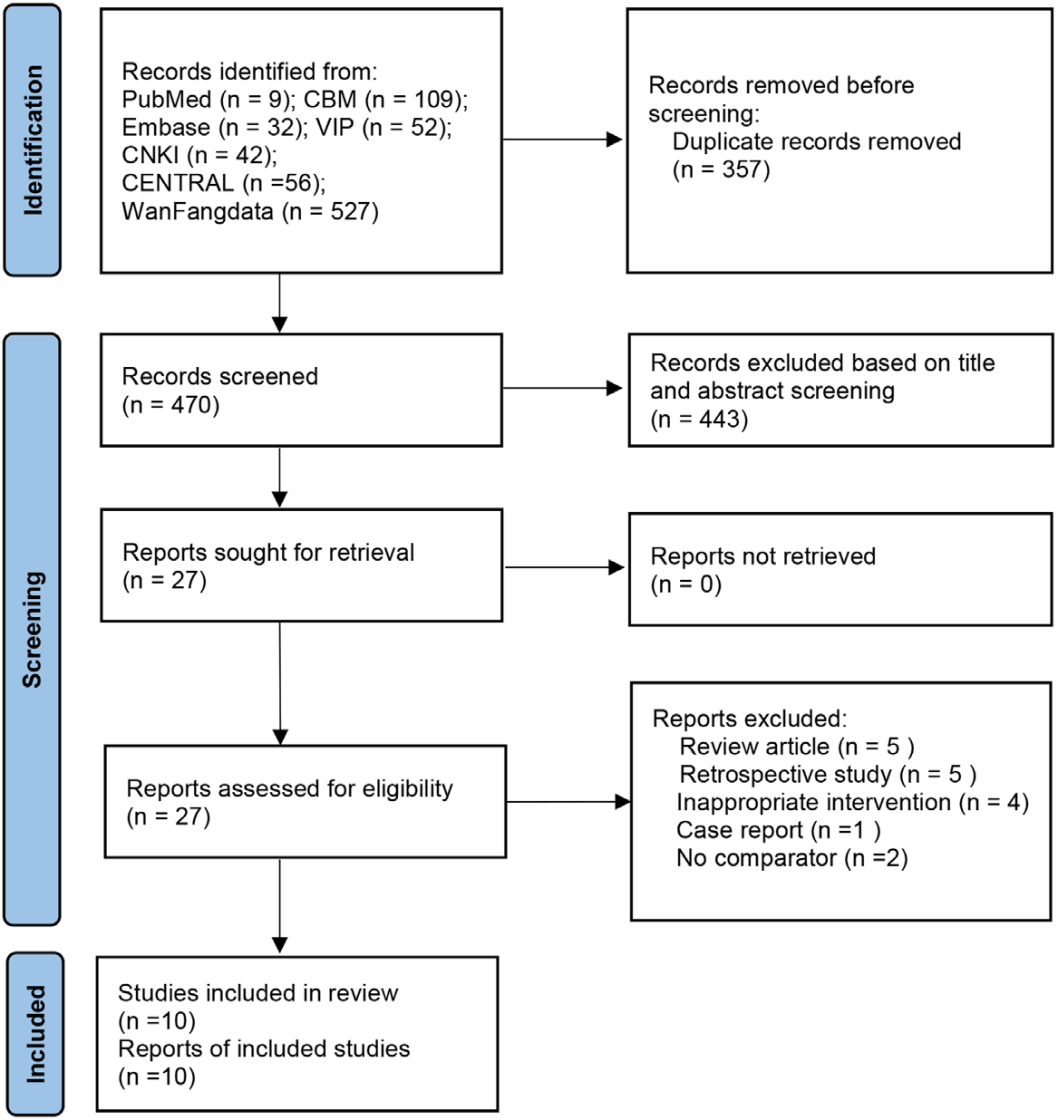
Literature selection flow diagram.

A total of 827 articles were searched in the literature on acupuncture for anxiety and depression in SCI patients, of which 357 were excluded due to duplicate publications and 443 were excluded through further screening by reading the titles and abstracts of the literature. Finally, further screening by reading the remaining literature in its entirety resulted in the inclusion of 10 studies^[13–22]^ based on the inclusion and exclusion criteria above. A flowchart of the selection process is shown in Figure 1.

### 3.2. The basic characteristics and quality evaluation included in the study

**Figure 2.**
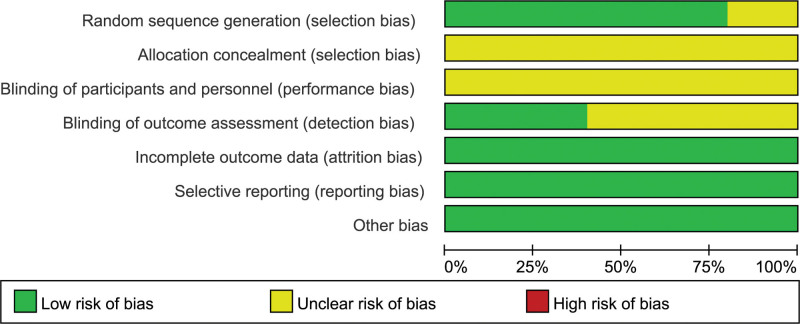
Risk of bias graph.

**Figure 3.**
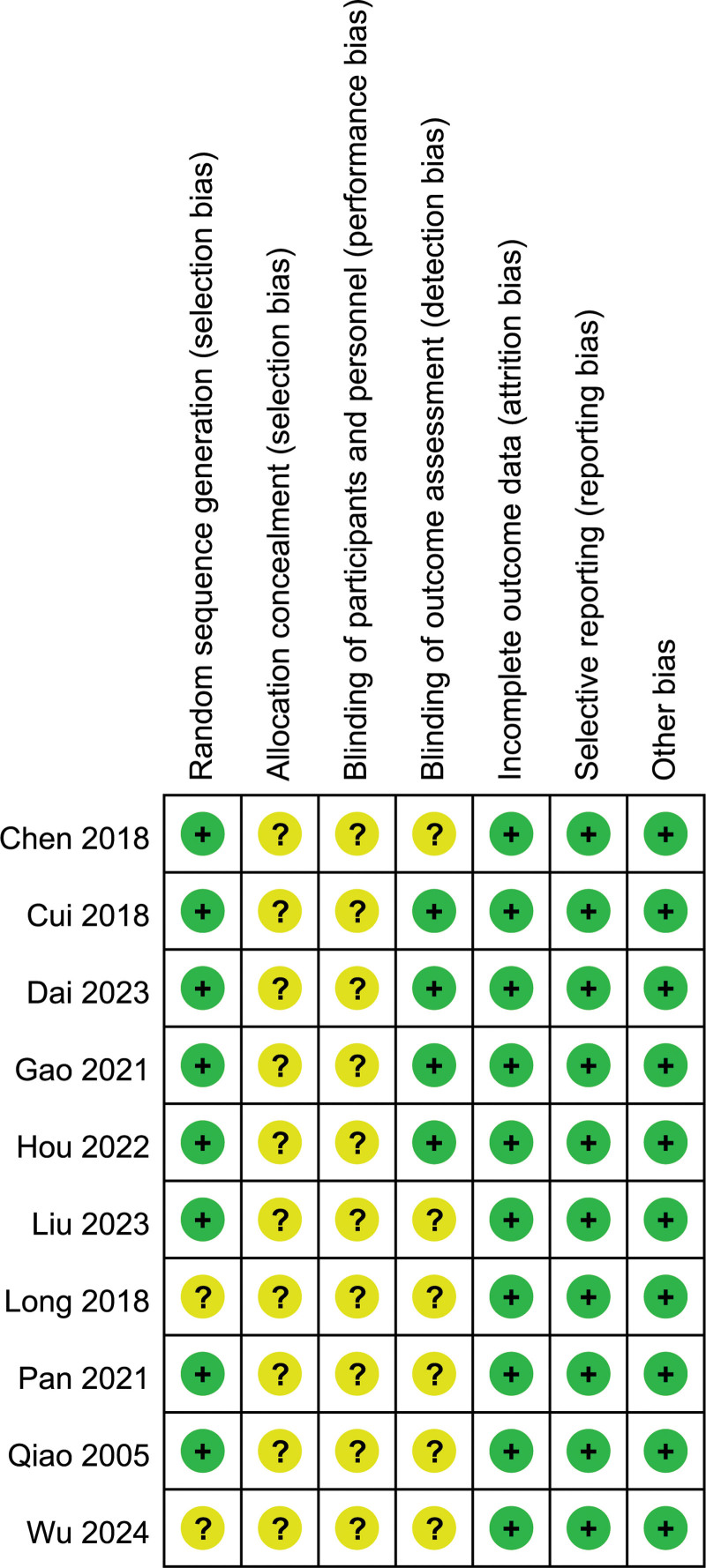
Risk of bias summary.

A total of 10 trials were included in this systematic review, with 361 cases in the experimental group and 355 cases in the control group. All studies had small sample sizes, ranging from 42 to 129 participants. The characteristics of the studies are shown in Table 1. Of the 10 studies included, 7 studies^[14–20]^ used a randomized numerical table method, 1 study^[13]^ used a stratified randomization method and 2 studies^[21,22]^ referred only to randomization, not to a specific method. None of the studies mention allocation concealment. Although some studies did not report blinding, it is unlikely that the removal of blinding would have affected the assessment of outcome indicators, as some outcome indicators require laboratory equipment. All included trials reported complete data for each of the primary outcome measures, including the number of lost visits and withdrawals and their causes. Their attrition bias was therefore assessed as low risk of bias. We did not identify any other potential risk of bias in the 10 RCTs. We therefore rated this as “low risk.” The risk of bias is shown in Figures 2 and 3.

**Table 1:** Characteristics of the included randomized clinical trials.

First author (yr)	Age (yr)		Gender (male/female)		Sample size		Intervention		Outcomes (time points for evaluation)
	EG	CG	EG	CG	EG	CG	EG	CG	
Chen (2018)	48.16 ± 5.12	47.98 ± 5.27	17/10	18/9	27	27	Electroacupuncture, conventional treatment	Conventional treatment	SAS (33 d); TER (33 d)
Cui et al (2018)	38 ± 12	37 ± 9	17/8	19/6	25	25	Electroacupuncture, conventional treatment	Conventional treatment	HAMD (30, 60 d); HAMA (30, 60 d); MBI (30, 60 d)
Dai (2023)	73.69 ± 2.31	73.54 ± 2.48	19/11	23/7	30	30	Electroacupuncture, conventional treatment	Conventional treatment	SAS (28 d); SDS (28 d); 5-HT (28 d); TNF-α (28 d); TER (28 d)
Gao et al (2021)	39.25 ± 12.10	38.86 ± 10.95	15/10	16/9	25	25	Acupuncture, conventional treatment	Conventional treatment	HAMD (84 d); HAMA (84 d); 5-HT(84 d)
Hou et al (2022)	39.20 ± 5.37	40.08 ± 7.29	37/20	34/22	57	56	Electroacupuncture, conventional treatment	Conventional treatment	SAS (28 d); SDS (28 d); 5-HT (28 d); TNF-α (28 d); TER (28 d)
Liu et al (2023)	46.290 ± 7.23	48.13 ± 6.12	19/9	16/12	28	28	Electroacupuncture, conventional treatment	Conventional treatment	SAS (28 d); SDS (28 d); MBI (28 d); TER (28 d)
Long et al (2018)	38.2 ± 2.8	37.5 ± 2.6	20/18	22/16	38	38	Acupuncture, conventional treatment	Conventional treatment	HAMD (28 d); HAMA (28 d); TER (28 d)
Pan et al (2021)	46.57 ± 4.25	46.83 ± 4.86	28/15	29/14	43	43	Electroacupuncture, conventional treatment	Conventional treatment	SAS (84 d); SDS (84 d); MBI (84 d)
Qiao et al (2005)	30 ± 11	32 ± 9	14/8	15/5	22	20	Electroacupuncture, conventional treatment	Conventional treatment	HAMD (42 d); TER (42 d)
Wu et al (2024)	39.48 ± 9.27	40.11 ± 8.69	41/25	40/23	66	63	Acupuncture, conventional treatment	Conventional treatment	HAMD (90 d); HAMA (90 d); 5-HT (90 d); MBI (90 d); TER (90 d)

5-HT = 5-hydroxytryptamine, CG = control group, EG = experimental group, HAMA = Hamilton Anxiety Scale, HAMD = Hamilton Depression Scale, MBI = Modified Barthel Index, SAS = Self-rating Anxiety Scale, SDS = Self-rating Depression Scale, TER = total effective rate, TNF-α = tumor necrosis factor-α.

### 3.3. Total clinical efficacy rate

**Figure 4.**
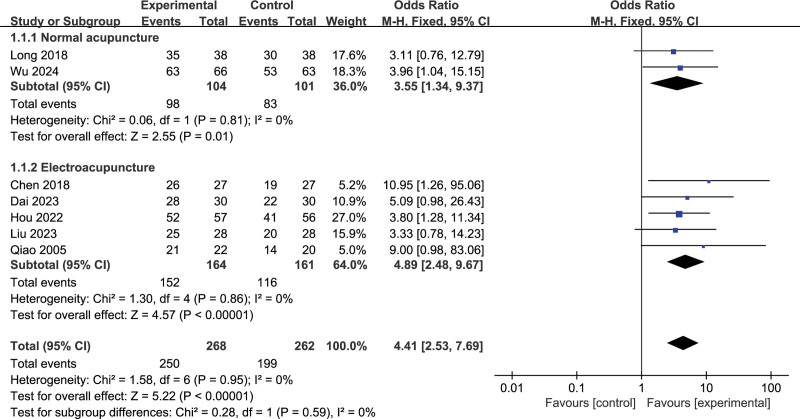
Meta-analysis and forest plot for total clinical efficacy rate.

A total of 7 studies^[14–17,20–22]^ reported a total clinical efficacy rate, of 268 cases in the experimental group (EG) and 262 cases in the control group (CG) overall. The results of the heterogeneity test showed that there was homogeneity between all the studies (*P* > .05, *I*^2^ = 0%), and a fixed effect model was applied. An overall meta-analysis showed that EG significantly improved the total clinical efficacy rate compared to CG (OR = 4.41 [95% CI: 2.53–7.69], *P* < .001). Based on different interventions, subgroup analyses were performed. The results showed that the combination of normal acupuncture and conventional treatment was superior to conventional treatment in improving the total clinical effective rate (OR = 3.55 [95% CI: 1.34–9.37], *P* < .001, *I*^2^ = 0%). In addition, subgroup analyses also showed that electroacupuncture combined with conventional therapy was superior to conventional therapy (OR = 4.89 [95% CI: 2.48–9.67], *P* < .001, *I*^2^ = 0%]. The results are shown in Figure 4.

### 3.4. Self-Rating Anxiety Scale

**Figure 5.**
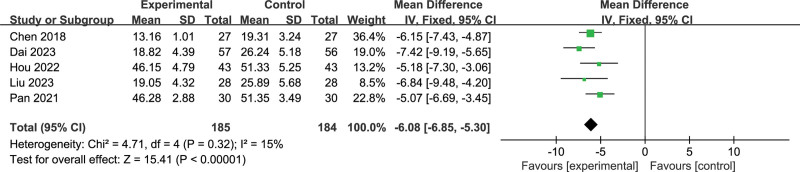
Meta-analysis and forest plot for Self-Rating Anxiety Scale.

Five studies^[15–18,20]^ reported the Self-Rating Anxiety Scale, with 185 participants in the EG and 184 participants in the CG overall. The results of the heterogeneity test showed that there was low heterogeneity between all studies (*P* > .05, *I*^2^ = 15%), and a fixed effect model was applied. An overall meta-analysis showed that EG significantly reduced Self-Rating Anxiety Scale compared to CG (MD = −6.08 [95% CI: −6.85 to −5.30], *P* < .001). The results are shown in Figure 5.

### 3.5. Self-Rating Depression Scale

**Figure 6.**
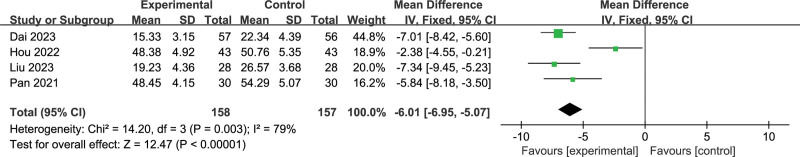
Meta-analysis and forest plot for Self-Rating Depression Scale.

Four studies^[16–18,20]^ reported the Self-Rating Depression Scale, with 158 participants in the EG and 157 participants in the CG overall. The results of the heterogeneity test showed that there was high heterogeneity between all studies (*P* < .01, *I*^2^ = 79%) and a random-effects model was applied. An overall meta-analysis showed that EG significantly reduced Self-Rating Depression Scale compared to CG (MD = −6.01 [95% CI: −6.95 to −5.07], *P* < .001). The results are shown in Figure 6.

### 3.6. Hamilton Depression Scale

**Figure 7.**
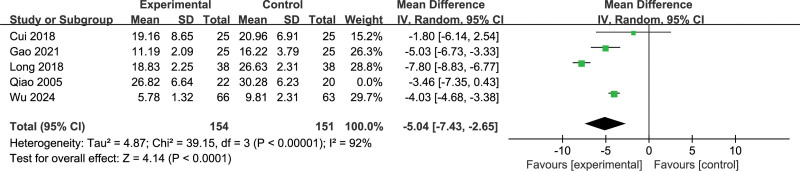
Meta-analysis and forest plot for Hamilton Depression Scale.

Five studies^[13,14,19,21,22]^ reported the Hamilton Depression Scale, with 154 participants in the EG and 151 participants in the CG overall. The results of the heterogeneity test showed that there was high heterogeneity between all studies (*P* < .01, *I*^2^ = 92%) and a random-effects model was applied. An overall meta-analysis showed that EG significantly reduced Hamilton Depression Scale compared to CG (MD = −5.04 [95% CI: −7.43 to −2.65], *P* < .001). The results are shown in Figure 7.

### 3.7. Hamilton Anxiety Scale

**Figure 8.**
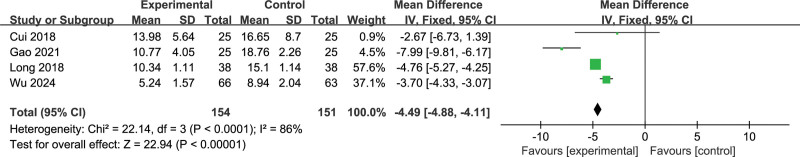
Meta-analysis and forest plot for Hamilton Anxiety Scale.

Four studies^[13,19,21,22]^ reported the Hamilton Anxiety Scale, with 154 participants in the EG and 151 participants in the CG overall. The results of the heterogeneity test showed that there was high heterogeneity between all studies (*P* < .01, *I*^2^ = 86%) and a random-effects model was applied. An overall meta-analysis showed that EG significantly reduced Hamilton Anxiety Scale compared to CG (MD = −4.49 [95% CI: −4.88 to −4.11], *P* < .001). The results are shown in Figure 8.

### 3.8. Modified Barthel Index

**Figure 9.**
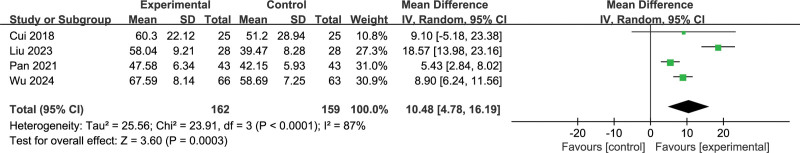
Meta-analysis and forest plot for Modified Barthel Index.

Four studies^[13,18,20,21]^ reported the Modified Barthel Index, with 162 participants in the EG and 159 participants in the CG overall. The results of the heterogeneity test showed that there was high heterogeneity between all studies (*P* < .01, *I*^2^ = 87%) and a random-effects model was applied. An overall meta-analysis showed that EG significantly improved MBI compared to CG (MD = 10.48 [95% CI: 4.78–16.19], *P* < .001). The results are shown in Figure 9.

### 3.9. 5-hydroxytryptamine

**Figure 10.**
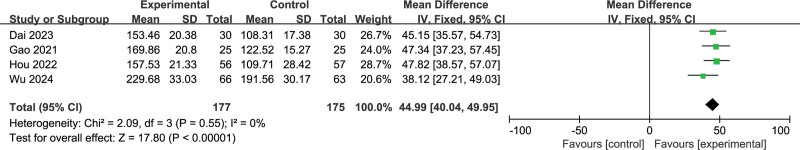
Meta-analysis and forest plot for 5-hydroxytryptamine.

Four studies^[16,17,19,21]^ reported 5-HT, with 177 participants in the EG and 175 participants in the CG overall. The results of the heterogeneity test showed that there was high heterogeneity between all studies (*P* > .05, *I*^2^ = 0%), and a random-effects model was applied. An overall meta-analysis showed that EG significantly improved 5-HT compared to CG (MD = 44.99 [95% CI: 40.04–49.95], *P* < .001). The results are shown in Figure 10.

### 3.10. Tumor necrosis factor-α

**Figure 11.**
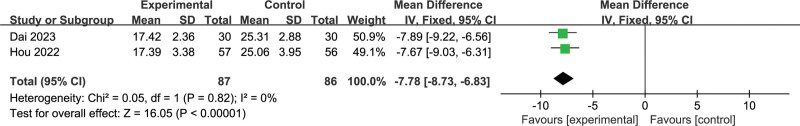
Meta-analysis and forest plot for tumor necrosis factor-α.

Two studies^[16,17]^ reported Tumor necrosis factor-α, with 87 participants in the EG and 86 participants in the CG overall. The results of the heterogeneity test showed that there was high heterogeneity between all studies (*P* > .05, *I*^2^ = 0%), and a random-effects model was applied. An overall meta-analysis showed that EG significantly reduced TNF-α compared to CG (MD = −7.78 [95% CI: −8.73 to −6.83], *P* < .001). The results are shown in Figure 11.

### 3.11. Sensitivity analyses

In this review, we performed sensitivity analyses for the main results by removing individual studies. The results showed that the summary analyses of the main results were stable (Table 2).

**Table 2:** Sensitivity analysis for total efficacy rate.

Outcomes	References	Weight	Effect size	95% CI	*P*	*I* ^2^
TER	Chen 2018	5.2%	4.05	2.27–7.23	<.001	0%
	Dai 2023	10.9%	4.32	2.39–7.82	<.001	0%
	Hou 2022	27.0%	4.63	2.42–8.86	<.001	0%
	Liu 2023	15.9%	4.61	2.52–8.44	<.001	0%
	Long 2018	17.6%	4.69	2.55–8.60	<.001	0%
	Qiao 2005	5.0%	4.17	2.34–7.42	<.001	0%
	Wu 2024	18.3%	4.51	2.44–8.32	<.001	0%

CI = confidence interval, TER = total clinical efficacy rate.

## 4. Discussion

This systematic review and meta-analysis assessed the efficacy of acupuncture for the treatment of anxiety and depression in SCI patients. Anxiety and depression are common psychological disorders in SCI, with a much higher probability of prevalence than in the general population, and increasing with disability and pain levels.^[23,24]^ Induction of anxiety and depression after SCI may be caused by these aspects. First, SCI consists of 2 phases, the first phase consists of the destruction of the structures associated with the nervous system, then in the second phase, SCI causes vascular destruction thereby allowing the invasion of immune cells into the spinal cord tissues leading to the release of inflammatory cytokines, this infiltration promotes neuronal inflammation and further causes secondary damage.^[25]^ Proinflammatory cytokines disrupt neural circuits involved in the pathogenesis of depression, decrease the bioavailability of neurotransmitters such as 5-HT during synaptic transmission, and increase the reuptake expression of 5-HT, which can lead to specific depressive symptoms,^[26]^ and may even interfere with or circumvent the efficacy of antidepressants.^[27]^

Currently, anxiety and depression are mainly treated with drugs, electroconvulsive, and psychological therapies, however, according to research findings, 70%–85.7% of patients experienced side effects of antidepressant drugs, and more than 52.29% of depressed patients had low adherence to antidepressant medications, with side effects such as insomnia, anxiety, dry mouth, nausea, tremor, and weight gain being the most common in the depressed population,^[28–30]^ long-term use is susceptible to drug resistance and potential reproductive toxicity effects.^[31]^ The psychotherapeutic process can also produce new anxiety and depression.^[32]^ Electroconvulsive therapy is a highly effective treatment for depressive episodes. However, it is prone to induce cognitive decline.^[33]^

Acupuncture, which has a history of more than 3000 years in China, is a non-pharmacological and safe traditional Chinese treatment that not only effectively improves depression after SCI,^[34,35]^ but also reduces the use of drugs and the side effects of medication.^[36,37]^ Clinical trials have proved that the efficacy of acupuncture is similar to that of conventional drug, and the effect is faster and better, especially for patients with mild to moderate depression, the first choice of acupuncture treatment is more effective.^[38,39]^ Among them, the governor channel is almost identical to the spinal cord alignment and location, and the Jiaji acupoint (EX-B2) and bladder channel are also symmetrically distributed on both sides of the spinal cord. In the present study, we found that acupuncture treatment was beneficial in improving 5-HT, reducing TNF-α compared to CG. Modern scientific research has also proved that acupuncture can achieve the effect of reducing depression in patients through a variety of complex pathways, for example, acupuncture can regulate multiple signaling pathways, improve the pathological changes in the hippocampus, and at the same time, make the pro-inflammatory factors decrease, increase neurotransmitters, inhibit the activation of hippocampal microglia and astrocytes to significantly alleviate the symptoms of depression.^[40–42]^ In addition, it modulates synaptic plasticity and enhances synaptic transmission,^[43,44]^ increases blood flow in the prefrontal and bilateral temporal cortices.^[45]^ It also improves pain memory, and exerts anxiolytic effects,^[46]^ ultimately helping to restore neurogenesis and improve depressive-like behaviors caused by chronic neuropathic pain, exerting a long-term antidepressant effect.^[47]^

Some limitations of this review are outlined below. First, all the studies were conducted in China, and the lack of studies from other countries and regions, as well as data from other populations, limits their applicability. Second, all types of SCI were selected for this review, which increases the heterogeneity of the results. The specific acupuncture points and frequency of intervention varied, which increases the difficulty of clinical application, and future clinical trials need to investigate the exact specific acupuncture points and frequency of intervention.

## 5. Conclusion

This study shows that acupuncture is beneficial in improving anxiety and depression, improving neurotransmitter levels in the brain, reducing serum inflammatory factors, and improving the ability to perform daily activities in patients with SCI. Further original and high-quality research is needed to verify the conclusions of this study.

## Author contributions

**Data curation:** Ke Liu, Xiaoyong Gao, Liang Ou, Le Zhang.

**Writing—original draft:** Ke Liu, Xiaoyong Gao.

**Writing—review & editing:** Ke Liu, Le Zhang, Jianjun Kuang.

**Methodology:** Xiaoyong Gao, Liang Ou.

**Software:** Xiaoyong Gao.

**Funding acquisition:** Liang Ou, Jianjun Kuang.

**Supervision:** Liang Ou, Zuyu Tang.

**Validation:** Liang Ou, Haoming Zhao, Sheng Hua.

**Visualization:** Yixiao Xiong.

## Supplementary Material


