# Supplementary material for: Acupuncture for the treatment of anxiety and depression in patients with spinal cord injury: A study protocol for systematic review and meta analysis
Source: Medicine (Baltimore). 2024 Sep 20;103(38):e39701. doi: 10.1097/MD.0000000000039701 (PMC11419492; doi:10.1097/MD.0000000000039701)
Supplement: Supplementary file 1 [file medi-103-e39701-s001.docx]

**Acupuncture for the Treatment of Anxiety and Depression in Patients with Spinal Cord Injury: A Systematic Review and Meta-Analysis**

**Ke Liu: First author, Hunan University of Chinese Medicine, Changsha 410208, Hunan, China**

**Supplementary Table 1. The search strategy in this review.**

**Search strategy for PubMed (9)**

| Search | Terms |
| --- | --- |
| #1 | (((((((((((Acupuncture Therapy[MeSH Terms]) OR (Acupuncture Treatment)) OR (Acupuncture Treatments)) OR (Treatment, Acupuncture)) OR (Therapy, Acupuncture)) OR (Pharmacoacupuncture Treatment)) OR (Treatment, Pharmacoacupuncture)) OR (Pharmacoacupuncture Therapy)) OR (Therapy, Pharmacoacupuncture)) OR (Acupotomy)) OR (Acupotomies)) OR (Acupuncture) |
| #2 | ((((((((((((((((((((((((((((((((((((((Spinal Cord Injuries[MeSH Terms]) OR (Injuries, Spinal Cord)) OR (Cord Injuries, Spinal)) OR (Cord Injury, Spinal)) OR (Injury, Spinal Cord)) OR (Spinal Cord Injury)) OR (Myelopathy, Traumatic)) OR (Myelopathies, Traumatic)) OR (Traumatic Myelopathies)) OR (Traumatic Myelopathy)) OR (Spinal Cord Trauma)) OR (Cord Trauma, Spinal)) OR (Cord Traumas, Spinal)) OR (Spinal Cord Traumas)) OR (Trauma, Spinal Cord)) OR (Traumas, Spinal Cord)) OR (Post-Traumatic Myelopathy)) OR (Myelopathies, Post-Traumatic)) OR (Myelopathy, Post-Traumatic)) OR (Post-Traumatic Myelopathies)) OR (Post Traumatic Myelopathy)) OR (Spinal Cord Contusion)) OR (Contusion, Spinal Cord)) OR (Contusions, Spinal Cord)) OR (Cord Contusion, Spinal)) OR (Cord Contusions, Spinal)) OR (Spinal Cord Contusions)) OR (Spinal Cord Laceration)) OR (Cord Laceration, Spinal)) OR (Cord Lacerations, Spinal)) OR (Laceration, Spinal Cord)) OR (Lacerations, Spinal Cord)) OR (Spinal Cord Lacerations)) OR (Spinal Cord Transection)) OR (Cord Transection, Spinal)) OR (Cord Transections, Spinal)) OR (Spinal Cord Transections)) OR (Transection, Spinal Cord)) OR (Transections, Spinal Cord) |
| #3 | ((((((((((((((((((((((((((((((((((((((((depression[MeSH Terms]) OR (Depressive Symptoms)) OR (Depressive Symptom)) OR (Symptom, Depressive)) OR (Emotional Depression)) OR (Depression, Emotional)) OR (Depressive Disorder)) OR (Depressive Disorders)) OR (Disorder, Depressive)) OR (Disorders, Depressive)) OR (Neurosis, Depressive)) OR (Depressive Neuroses)) OR (Depressive Neurosis)) OR (Neuroses, Depressive)) OR (Depression, Endogenous)) OR (Depressions, Endogenous)) OR (Endogenous Depression)) OR (Endogenous Depressions)) OR (Melancholia)) OR (Melancholias)) OR (Unipolar Depression)) OR (Depression, Unipolar)) OR (Depressions, Unipolar)) OR (Unipolar Depressions)) OR (Depressive Syndrome)) OR (Depressive Syndromes)) OR (Syndrome, Depressive)) OR (Syndromes, Depressive)) OR (Depression, Neurotic)) OR (Depressions, Neurotic)) OR (Neurotic Depression)) OR (Neurotic Depressions)) OR (anxiety)) OR (psychology)) OR (mentality)) OR (worry)) OR (anxious)) OR (depressed)) OR (gloomy)) OR (despondent)) OR (mood) |
| #4 | ((randomized controlled trial[Publication Type]) OR (randomized[Title/Abstract])) OR (placebo[Title/Abstract]) |
| #5 | #1 AND #2 AND #3 AND #4 |

**Search strategy for Embase (32)**

| Search | Terms |
| --- | --- |
| #1 | 'acupuncture'/exp OR 'acupuncture' OR 'acupuncture treatment' OR 'acupuncture treatments' OR 'treatment, acupuncture' OR 'therapy, acupuncture' OR 'pharmacoacupuncture treatment' OR 'treatment, pharmacoacupuncture' OR 'pharmacoacupuncture therapy' OR 'therapy, pharmacoacupuncture' OR 'acupotomy' OR 'acupotomies' |
| #2 | 'spinal cord injury'/exp OR 'spinal cord injury' OR 'injuries, spinal cord' OR 'cord injuries, spinal' OR 'cord injury, spinal' OR 'injury, spinal cord' OR 'myelopathy, traumatic' OR 'myelopathies, traumatic' OR 'traumatic myelopathies' OR 'traumatic myelopathy' OR 'spinal cord trauma' OR 'cord trauma, spinal' OR 'cord traumas, spinal' OR 'spinal cord traumas' OR 'trauma, spinal cord' OR 'traumas, spinal cord' OR 'post-traumatic myelopathy' OR 'myelopathies, post-traumatic' OR 'myelopathy, post-traumatic' OR 'post-traumatic myelopathies' OR 'post traumatic myelopathy' OR 'spinal cord contusion' OR 'contusion, spinal cord' OR 'contusions, spinal cord' OR 'cord contusion, spinal' OR 'cord contusions, spinal' OR 'spinal cord contusions' OR 'spinal cord laceration' OR 'cord laceration, spinal' OR 'cord lacerations, spinal' OR 'laceration, spinal cord' OR 'lacerations, spinal cord' OR 'spinal cord lacerations' OR 'spinal cord transection' OR 'cord transection, spinal' OR 'cord transections, spinal' OR 'spinal cord transections' OR 'transection, spinal cord' OR 'transections, spinal cord' |
| #3 | 'depression'/exp OR 'depression' OR 'depressive symptoms' OR 'depressive symptoms' OR 'depressive symptom' OR 'depressive symptom' OR 'symptom, depressive' OR 'emotional depression' OR 'depression, emotional' OR 'depressive disorder' OR 'depressive disorder' OR 'depressive disorders' OR 'disorder, depressive' OR 'disorders, depressive' OR 'neurosis, depressive' OR 'depressive neuroses' OR 'depressive neurosis' OR 'depressive neurosis' OR 'neuroses, depressive' OR 'depression, endogenous' OR 'depression, endogenous' OR 'depressions, endogenous' OR 'endogenous depression' OR 'endogenous depressions' OR 'melancholia' OR 'melancholia' OR 'melancholias' OR 'unipolar depression' OR 'depression, unipolar' OR 'depression, unipolar' OR 'depressions, unipolar' OR 'unipolar depressions' OR 'depressive syndrome' OR 'depressive syndrome' OR 'depressive syndromes' OR 'syndrome, depressive' OR 'syndromes, depressive' OR 'depression, neurotic' OR 'depression, neurotic' OR 'depressions, neurotic' OR 'neurotic depression' OR 'neurotic depressions' OR 'anxiety' OR 'anxiety' OR 'psychology' OR 'psychology' OR 'mentality' OR 'worry' OR 'worry' OR 'anxious' OR 'depressed' OR 'gloomy' OR 'despondent' OR 'mood' |
| #4 | 'clinical trial'/exp OR 'clinical trial' OR (('clinical' OR 'clinical'/exp OR clinical) AND ('trial' OR 'trial'/exp OR trial)) OR (randomized AND controlled AND ('trial'/exp OR trial)) OR trials |
| #5 | #1 AND #2 AND #3 AND #4 |

**Search strategy for CENTRAL (56)**

| Search | Terms |
| --- | --- |
| #1 | MeSH descriptor: [Acupuncture Therapy] explode all trees |
| #2 | (Acupuncture Treatment) OR (Acupuncture Treatments) OR (Treatment, Acupuncture) OR (Therapy, Acupuncture) OR (Pharmacoacupuncture Treatment) OR (Treatment, Pharmacoacupuncture) OR (Pharmacoacupuncture Therapy) OR (Therapy, Pharmacoacupuncture) OR (Acupotomy) OR (Acupotomies) OR (Acupuncture) |
| #3 | MeSH descriptor: [Spinal Cord Injuries] explode all trees |
| #4 | (Injuries, Spinal Cord) OR (Cord Injuries, Spinal) OR (Cord Injury, Spinal) OR (Injury, Spinal Cord) OR (Spinal Cord Injury) OR (Myelopathy, Traumatic) OR (Myelopathies, Traumatic) OR (Traumatic Myelopathies) OR (Traumatic Myelopathy) OR (Spinal Cord Trauma) OR (Cord Trauma, Spinal) OR (Cord Traumas, Spinal) OR (Spinal Cord Traumas) OR (Trauma, Spinal Cord) OR (Traumas, Spinal Cord) OR (Post-Traumatic Myelopathy) OR (Myelopathies, Post-Traumatic) OR (Myelopathy, Post-Traumatic) OR (Post-Traumatic Myelopathies) OR (Post Traumatic Myelopathy) OR (Spinal Cord Contusion) OR (Contusion, Spinal Cord) OR (Contusions, Spinal Cord) OR (Cord Contusion, Spinal) OR (Cord Contusions, Spinal) OR (Spinal Cord Contusions) OR (Spinal Cord Laceration) OR (Cord Laceration, Spinal) OR (Cord Lacerations, Spinal) OR (Laceration, Spinal Cord) OR (Lacerations, Spinal Cord) OR (Spinal Cord Lacerations) OR (Spinal Cord Transection) OR (Cord Transection, Spinal) OR (Cord Transections, Spinal) OR (Spinal Cord Transections) OR (Transection, Spinal Cord) OR (Transections, Spinal Cord) |
| #5 | MeSH descriptor: [Depression] explode all trees |
| #6 | (Depressive Symptoms) OR (Depressive Symptom) OR (Symptom, Depressive) OR (Emotional Depression) OR (Depression, Emotional) OR (Depressive Disorder) OR (Depressive Disorders) OR (Disorder, Depressive) OR (Disorders, Depressive) OR (Neurosis, Depressive) OR (Depressive Neuroses) OR (Depressive Neurosis) OR (Neuroses, Depressive) OR (Depression, Endogenous) OR (Depressions, Endogenous) OR (Endogenous Depression) OR (Endogenous Depressions) OR (Melancholia) OR (Melancholias) OR (Unipolar Depression) OR (Depression, Unipolar) OR (Depressions, Unipolar) OR (Unipolar Depressions) OR (Depressive Syndrome) OR (Depressive Syndromes) OR (Syndrome, Depressive) OR (Syndromes, Depressive) OR (Depression, Neurotic) OR (Depressions, Neurotic) OR (Neurotic Depression) OR (Neurotic Depressions) OR (anxiety) OR (psychology) OR (mentality) OR (worry) OR (anxious) OR (depressed) OR (gloomy) OR (despondent) OR (mood) |
| #7 | (#1 OR #2) AND (#3 OR #4) AND (#5 OR #6) |

**Search strategy for CNKI（42）**

(SU%=针刺 + 针 + 针灸) AND (SU%=脊髓损伤 + 截瘫 + 脊髓) AND (SU%=焦虑 + 抑郁)

**Search strategy for WanFang（527）**

主题: ("针刺" OR "针"OR "针灸") and 主题: ("脊髓损伤" OR"截瘫" OR"脊髓") and 主题: ("焦虑"OR"抑郁")

**Search strategy for VIP（52）**

(U=针刺 OR 针 OR 针灸) AND (U=脊髓损伤 OR 截瘫 OR 脊髓) AND (U=焦虑 OR 抑郁)

**Search strategy for CBM（109）**

("针刺"[全部字段:智能] OR "针"[全部字段:智能] OR "针灸"[全部字段:智能]) AND ("脊髓损伤"[全部字段:智能] OR "截瘫"[全部字段:智能] OR "截瘫"[全部字段:智能])AND ("焦虑"[全部字段:智能] OR "抑郁"[全部字段:智能])
